# A nine month old child with retropharyngeal abscess secondary to mastoid abscess presenting as torticollis: a case report

**DOI:** 10.4076/1757-1626-2-6460

**Published:** 2009-07-21

**Authors:** Janardhan Mydam, Prakash Thiagarajan

**Affiliations:** 1 Department of Pediatrics, Glan Clwyd Hospital BodelwyddanRhyl, Denbighshire, LL18 5UJUK; 2 Department of Pediatrics, Nobles HospitalIsle of Man, IM4 4RJUK

## Abstract

We describe the unusual case of a 9-month-old Caucasian girl with a retropharyngeal abscess secondary to a mastoid abscess who presented with torticollis. The retropharyngeal abscess was caused by pus from the mastoid abscess tracking down under the petrous part of the temporal bone to reach the retropharyngeal space via the fossa of Rosenmüller. We believe that this is the first time that this route of infection has been reported. The patient was treated successfully with 7 days of intravenous ceftriaxone and flucloxacillin followed by oral co-amoxiclav for 7 days, followed by physiotherapy.

## Introduction

Acute retropharyngeal abscess is usually seen in children under 4-5 years of age. The retropharyngeal space lies behind the pharynx between the buccopharyngeal fascia, which covers the constrictor muscle, and the prevertebral fascia. It extends from the base of the skull to the tracheal bifurcation. The space is divided into two lateral compartments (the space of Gillette) by a fibrous raphe. Each lateral space contains retropharyngeal nodes. As the retropharyngeal space communicates with the parapharyngeal space and the posterior mediastinum, any infection within the retropharyngeal space can spread to these areas [1]. Early detection and treatment of retropharyngeal infections is therefore vital.

In adults, retropharyngeal abscesses are usually caused by direct infection resulting from the presence of a foreign body, or from some kind of penetrating injury to the posterior pharyngeal wall. Naso-oropharyngeal infection is rarely responsible for the development of a retropharyngeal abscess in adults, as the lymph nodes in the retropharyngeal space usually disappear after the age of 4-5 years [1].

In children, retropharyngeal abscess is commonly caused by suppuration of the retropharyngeal lymph nodes, which drain from the nasopharynx, oropharynx, and tonsils. Rarely, retropharyngeal abscesses in children might also result from mastoid infection. Mastoiditis can produce intracranial and extracranial consequences. Intracranial complications include meningitis, sigmoid sinus thrombosis, and intracranial abscess, while extracranial complications include osteomyelitis, facial nerve palsy, suppurative labyrinthitis, subperiosteal and zygomatic abscesses, and abscesses in the deep neck (e.g. parapharyngeal and retropharyngeal) spaces [2].

The most common mechanism by which a mastoid abscess gives rise to a retropharyngeal abscess involves erosion of the mastoid tip, along with the tracking of pus-via the sheath of either the digastric muscle or the sternocleidomastoid muscle-into the retropharyngeal space through the parapharyngeal space [2]. Here, however, we describe the first reported case of a retropharyngeal abscess resulting from a mastoid abscess via a mechanism that most likely involved erosion of the mastoid tip and tracking of pus under the surface of the petrous part of the temporal bone, so that it reached the retropharyngeal space through the fossa of Rosenmüller.

## Case presentation

A 9-month-old Caucasian girl was referred by a health visitor to our centre with a 6-week history of being unable to look upwards, constant neck flexion, and deviation to the right side. When distracted while sitting, the child would turn her whole body around instead of turning just her neck to look for the source of the distraction. She had no history of ear discharge, swallowing difficulties, tuberculosis, or trauma, and she had no significant past medical illness or history. She was not receiving any treatment or medication.

On examination, the child was active, alert and afebrile. Her weight was 8.6 kg (25^th^-50^th^ percentile; height unknown). She kept her neck persistently flexed, with right-sided torticollis. Passive movements of the neck were restricted. There was tenderness over the left mastoid process, but no tenderness along the sternocleidomastoid muscle. There were a few small, discrete, non-tender, freely mobile lymph nodes palpable on both sides of the neck. No obvious abscess was palpated in the neck, and there were no other signs of inflammation. The carotid arteries were palpable and normal. Otoscopic examination revealed normal tympanic membranes, and there was no discharge of pus from either of the ears. Examination of the throat showed a slight bulging of the posterior pharyngeal wall on the left side; the tonsils appeared normal. The remainder of the physical examination was normal. A clinical diagnosis of acquired non-dystonic torticollis was made.

**Figure 1.**
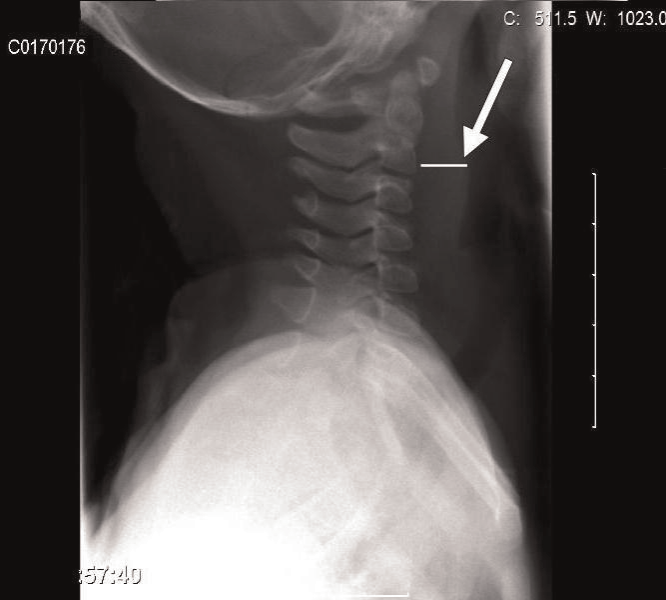
Plain radiograph lateral view of the neck taken at time of presentation showing retropharyngeal abscess.

**Figure 2.**
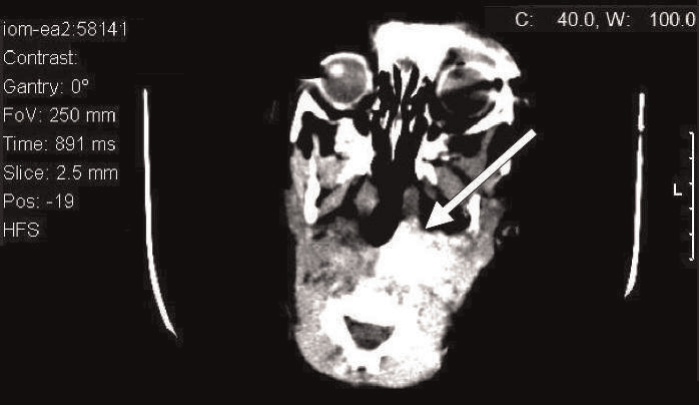
Axial computed tomography scan of the head and neck taken at time of presentation showing retropharyngeal abscess.

**Figure 3.**
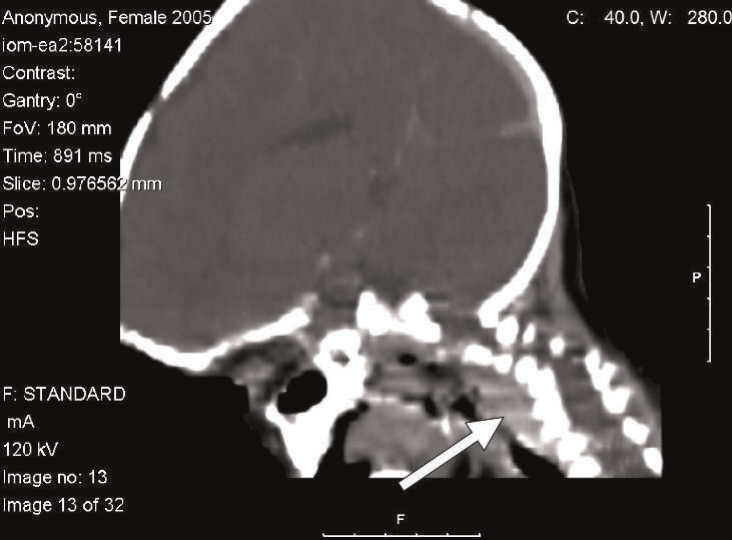
The sagittal reformation of the axial computed tomography scans (taken at time of presentation) show a retropharyngeal abscess that has tracked down from the mastoid abscess.

**Figure 4.**
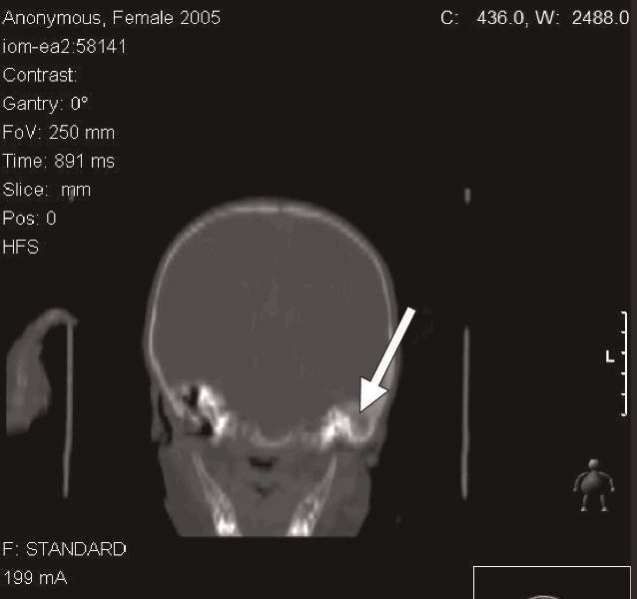
Coronal computed tomography scan of the head and neck taken at time of presentation using bone window settings, showing mastoid abscess on the left side.

**Figure 5.**
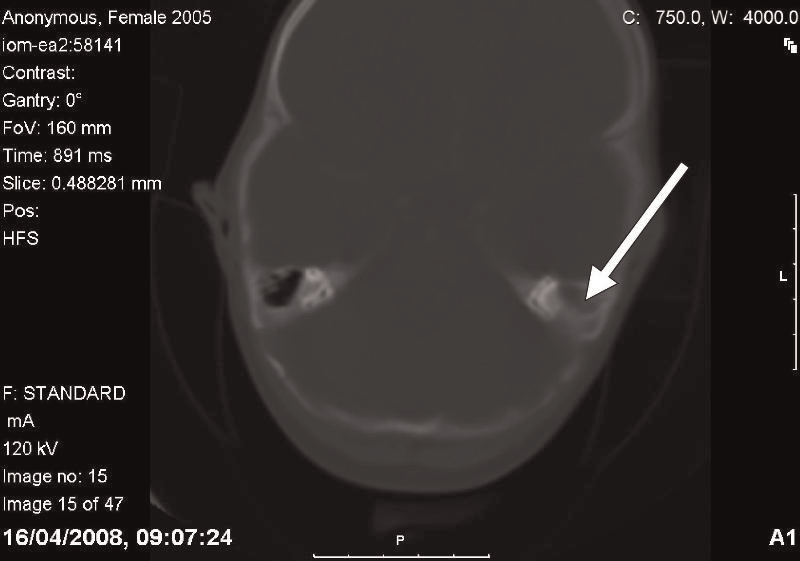
Axial computed tomography scan of the head and neck (taken at time of presentation) through the petrous bones shows osteolysis of the mastoid on the left side, indicating chronic mastoiditis with abscess.

Further investigations, including full blood count and tests for urea, electrolytes, and C-reactive protein, were all within the normal ranges. Glandular fever screening and the Mantoux test were both negative. An X-ray of the neck showed enlarged retropharyngeal and posterior tracheal spaces (Figure 1), while ultrasonography of the neck confirmed cervical lymphadenopathy and indicated the presence of inflammatory changes to the lymph nodes. A computed tomography (CT) scan of the head and neck showed a soft-tissue swelling on the left posterior aspect of the pharynx, which was enhanced on contrast-enhanced images in keeping with the infective pathology (Figures 2 and 3). There was also evidence of left acute mastoiditis, with large defects in the mastoid air cells measuring 9.9 mm in size. These defects were suggestive of a small abscess (Figures 4 and 5). The parapharyngeal spaces were normal.

**Figure 6.**
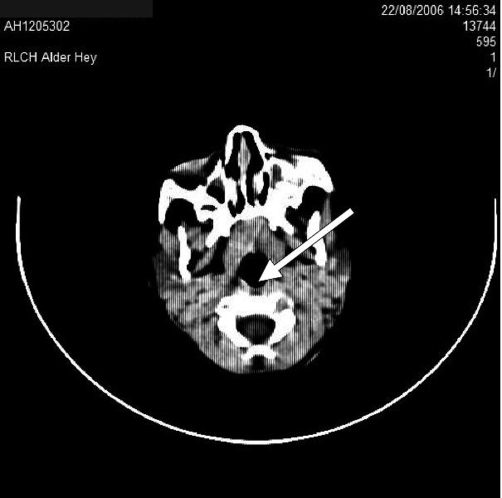
Axial computed tomography scan of the head and neck taken approximately 4 weeks after treatment initiation showing complete resolution of retropharyngeal abscess.

The patient was diagnosed with a retropharyngeal abscess secondary to an acute mastoid abscess. Blood cultures were negative. She was admitted to the High Dependency Unit (HDU), and treatment with intravenous ceftriaxone 80 mg/kg/q.d. and intravenous flucloxacillin 50 mg/kg/q.i.d. was initiated. The Ear, Nose and Throat (ENT) and anaesthetic teams were informed and warned of the possibility of an emergency situation. As the child was stable, medical management continued with regular reviews. Over the next 72 hours, her neck movements gradually improved. Intravenous antibiotics were continued for 7 days, during which time considerable clinical improvement was observed. Physiotherapy was initiated on day 7 to improve the range of neck movements, and the intravenous antibiotics were changed to oral co-amoxiclav for a further 7 days. After 15 days, the patient’s clinical condition had returned to normal. She was then referred to a regional tertiary pediatric ENT department, where our diagnosis of retropharyngeal abscess secondary to mastoid abscess was confirmed. Four weeks after the initiation of treatment, the patient underwent an additional CT scan, which showed complete resolution of the previously observed abnormalities (Figure 6).

## Discussion

We describe an unusual case in which mastoiditis resulted in the rare complication of retropharyngeal abscess in a 9-month-old girl. To our knowledge, there are no other reported cases of children with mastoiditis spreading to the retropharyngeal space.

We believe it is highly unlikely that the retropharyngeal abscess was the primary infection in our patient. Generally, the clinical course of a retropharyngeal abscess includes fever, neck swelling, drooling, and swallowing difficulties, and is typically associated with prior upper respiratory tract infection and a history of trauma caused by foreign bodies [3]. As the abscess grows, it usually causes significant respiratory distress, particularly in young children [4]. Our patient, however, displayed none of these classical presentations and did not experience any respiratory distress. This suggests that a retropharyngeal abscess was not present when her symptoms began.

Mastoiditis is likely to have been the primary source of infection in this patient. The chronic nature of mastoid infection, as evidenced by the CT scan showing osteolysis in the mastoid process (Figure 5), is consistent with the 6-week duration of symptoms in our patient. In contrast, the patient’s retropharyngeal abscess was limited to the left half of the retropharyngeal space and was not associated with any of the complications that normally result from retropharyngeal abscess (e.g. respiratory distress and swallowing difficulties). This suggests that it had a recent onset that was most likely caused by the spread of infection from the already-present mastoid abscess.

The most common routes by which pus normally spreads from the mastoid abscess are via the sheath of either the sternocleidomastoid or digastric muscle. The spreading of pus from a mastoid abscess via the sternocleidomastoid sheath results in the development of a Bezold’s abscess; if pus spreads via the digastric sheath, then a Citelli’s abscess results. Both forms of abscess can cause a parapharyngeal abscess and, subsequently, a retropharyngeal abscess due to the close proximity of these spaces to each other. In our case, however, physical examination and CT scans failed to show any sign of infection in the sternocleidomastoid or digastric muscles, suggesting that neither a Bezold’s abscess nor a Citelli’s abscess was the cause of our patient’s retropharyngeal abscess. Petrositis or suppuration of the lymph nodes around the internal jugular vein can also cause a retropharyngeal abscess secondary to a mastoid abscess. However, there were no signs of petrositis (which is normally associated with severe retrobulbar pain, diplopia, orbital cellulitis, and facial nerve palsy [2]) in our patient, suggesting that this was also an unlikely cause of her retropharyngeal abscess. Neither suppuration of lymph nodes around the internal jugular vein nor internal jugular vein thrombophlebitis [2] was considered responsible for the retropharyngeal abscess, as there was no evidence of thrombophlebitis.

Rarely, pus from a mastoid abscess can trickle down the petrous part of the temporal bone to reach the retropharyngeal space via the fossa of Rosenmüller. Although this route has been postulated as a possible method by which pus from a mastoid abscess can be spread [1], such a phenomenon has not been reported to date. Nonetheless, we believe that it is the most likely scenario in our patient. Blood cultures were negative; however, previous reports suggest that mastoiditis and retropharyngeal abscess are most commonly caused by *Streptococcus pneumoniae* and *Staphylococcus aureus* infections [3,5].

Retropharyngeal abscess can present as torticollis in 67% of cases due to spasm of the neck muscles [3]. Indeed, a recently published report described a rare case of retropharyngeal abscess complicated by torticollis in a 4-year-old girl [6]. The presenting symptoms of torticollis and the bulging of the posterior pharyngeal wall in our patient were consistent with clinical features found in an earlier series of children with retropharyngeal abscesses [3]. Retropharyngeal abscesses can also severely obstruct the airway; Zafereo and Pereira describe a child who presented with severe obstructive sleep apnea due to oropharyngeal narrowing caused by a retropharyngeal abscess [7]. However, our patient showed no signs of airway obstruction or mediastinitis.

When establishing a diagnosis of retropharyngeal abscess, the lateral X-ray is helpful, but the CT scan has enormous value as a diagnostic tool that can detect an abscess and help to establish a treatment regimen [8]. Courtney et al reviewed the medical records of 24 children with retropharyngeal abscesses and reported that the CT scan had a 75% accuracy for correctly identifying the abscess [9].

In terms of treatment, Vázquez López et al [10] suggest that the decision of whether to apply medical or surgical management depends on several factors, including the general condition of the child, the size of the abscess, potential complications arising from the abscess, and surgical accessibility. A number of other authors have suggested using surgery only when patients do not respond to medical treatment (e.g. intravenous antibiotics) [8,9,11]. We agree with this suggestion. A recent article has also identified a number of predictive factors that may be useful in identifying those children with retropharyngeal abscesses who should be treated with intravenous antibiotics alone [12]. As our patient’s abscess was restricted to the retropharyngeal region, and as the child was clinically stable without respiratory compromise, we chose medical management over surgical removal. The child was admitted to the HDU and observed closely, with her condition being reviewed at frequent intervals. As she showed definite signs of improvement after 48-72 hours, medical treatment was continued for a total of 2 weeks.

## Conclusions

Mastoiditis is a very rare cause of retropharyngeal abscess, involving the tracking of pus under the surface of the petrous part of the temporal bone. The diagnosis of retropharyngeal abscess should be considered in a child who presents with non-dystonic torticollis, as this is one of the most common clinical features of retropharyngeal abscess. Lateral X-ray is helpful in establishing a diagnosis, but CT is even more useful - not only for diagnostic purposes, but also for determining the extent of the abscess. In turn, this facilitates the selection of an appropriate treatment and management regimen. The decision of whether to use surgical or medical management depends on a variety of factors, including the patient’s general condition, the size of the abscess, and potential complications of both the mastoid and retropharyngeal abscesses. In a child who is generally well without any abscess-related complications, we would recommend medical management under intensive care, with frequent reviewing of the patient’s clinical condition.

## Abbreviations

CT: computed tomography
ENT: Ear Nose and Throat
HDU: high dependency unit
q.i.d: four times daily
